# NuRse-led home CardiovErsion for control of atrial fibrillation—RACE 6

**DOI:** 10.1007/s12471-025-01972-1

**Published:** 2025-07-31

**Authors:** Geert Hengstman, Salah A. M. Saïd, Ramon de Nooijer, Paul J. M. Voorhorst, Frank H. J. van der Holst, Francisca F. Kamphuis-Wolters, Isabelle C. Van Gelder, Harry J. G. M. Crijns

**Affiliations:** 1 Ambulance Oost, Hengelo, The Netherlands; 2 Department of Cardiology, Ziekenhuis Groep Twente, Hengelo, The Netherlands; 3 Department of Anaesthesiology, Ziekenhuis Groep Twente, Hengelo, The Netherlands; 4 Department of Cardiology, University Medical Centre Groningen, Groningen, The Netherlands; 5 Department of Cardiology and CARIM, Maastricht University Medical Centre, Maastricht, The Netherlands

**Keywords:** Atrial fibrillation, Electrical cardioversion, Shifting care, Hospital-at-home, Emergency care practitioner, Conscious sedation

## Abstract

**Background:**

Hospital care shifted to home may contribute to sustainability of health care. It is uncertain if home-based electrical cardioversion (ECV) is feasible.

**Methods:**

RACE‑6 is a prospective proof-of-concept pilot study on feasibility of ECV of persistent symptomatic atrial fibrillation (AF) at patient’s homes. It is performed by a mobile nurse-led team, including an emergency care practitioner (ECP) and a sedation nurse, and is remotely supervised by a cardiologist with an ambulance (driver) standby. To ensure safe ECV, the ECP assessed the patients’ homes beforehand for accessibility, hygiene, adequate space and light, electrical interference and explosive sources, electricity network stability, and the presence of an adequate informal caregiver overnight.

**Results:**

Six consenting patients with an uneventful previous in-hospital ECV for persistent AF developed one or two symptomatic recurrences and underwent in total 8 separate ECV attempts under conscious sedation at their homes. In all patients sinus rhythm returned and there were no early or late complications. Patients invariably preferred home cardioversion over cardioversion in-hospital.

**Discussion:**

Although applied in highly selected patients, home cardioversion may be extended to a wider selection of patients with persistent AF or even to patients with paroxysmal AF in need of acute restoration of sinus rhythm. Shortening time to cardioversion and early restoration of sinus rhythm may enhance patients’ quality of life and postpone AF progression. Home cardioversion may appear safe and improve cost-effectiveness of care but randomized controlled trials are needed to show that home cardioversion may keep AF patients out of the hospital and contribute to the sustainability of health care.

**Conclusion:**

Cardioversion at home is feasible and is generally well received by patients.

## What is new?

*What is known: *there are no data on cardioversion for atrial fibrillation (AF) at patients’ homes. Transmural management involving specialised nurses in Ambulance Services and hospital medical specialists have generally improved patient care.

*What is new: *this proof-of-concept pilot study shows that in highly selected cardioversion at home led by an emergency care practitioner, supported by a sedation nurse, with remote supervision by a cardiologist and a safety net ambulance present at the scene, is feasible. Patients invariably prefer home cardioversion over their experience with previous cardioversion in hospital.

*What are the perspectives: *home cardioversion may reduce hospital costs of care, shorten time in atrial fibrillation and hence reduce symptoms and prevent progression of AF. Safety remains to be established. If safety is proven, the indication for home cardioversion may be significantly broadened. The study may set an example not only for Netherlands healthcare but also Europe wide.

## Background

Electrical cardioversion (ECV) is widely used in patients with atrial fibrillation (AF) [1–3]. Elective procedures are performed in-hospital under adequate sedation and rhythm monitoring, and are mostly performed as day cases by cardiologist-supervised specialised nurses [4–7]. However, waiting times may be long, and hospital facilities are expensive. Moreover, in the Netherlands, hospital capacity is under considerable pressure due to a growing demand, complexity, and the cost of clinical care in an era of limited financial and human resources. Transforming health care is important, and re-organising tasks and responsibilities within local healthcare networks might contribute to a meaningful transformation [8]. In the Netherlands, it is legally regulated that emergency care practitioners (ECP) have the independent authority to perform reserved actions, such as electrical cardioversion of atrial fibrillation (Wet BIG artikel 36a). RACE‑6 is a proof-of-concept study [9] describing ECV for AF at patients’ homes, performed by a mobile ECP-led team, remotely supervised by a cardiologist.

## Methods

This prospective study was initiated by the Ambulance Service Oost, Hengelo, in collaboration with the Cardiology Department of Ziekenhuis Groep Twente Hospital, Almelo, all located in the Netherlands (NL). The study was approved by the Institutional Review Board of the Maastricht University Medical Centre, as well as the Dutch Healthcare Inspection, and was registered at ToetsingOnLine, NL42344.068.12.

The on-site mobile cardioversion team consisted of an ECP (ambulance ECPs are nurse practitioners with a background in intensive care, coronary care, or emergency departments, and have years of experience in ambulance care), a sedation nurse and an ambulance driver. The ECPs belonged to a specialised group of 2 officers and all ambulance drivers within Ambulance Service Oost. The group of sedation nurses consisted of 3 officers and were made available by the Ziekenhuis Groep Twente Hospital. The ECP was 24/7 available for calls from patients reporting recurrences. In case of a reported recurrence, home cardioversion was planned taking into account the overall service schedule, with the aim of performing cardioversion as soon as possible. Because eligible patients were symptomatic, cardioversion was preferably scheduled on the same day.

**Fig. 1:**
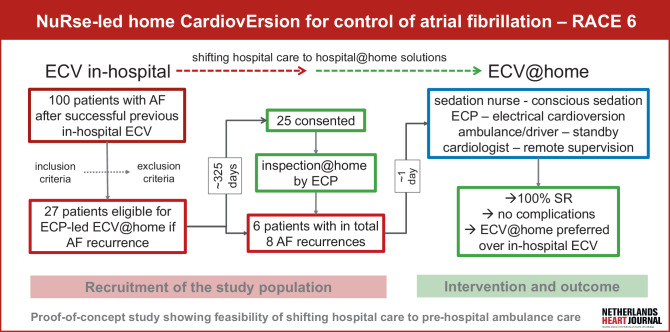
Infographic. The figure shows the study procedures, flow of patients, distribution of tasks among attending care officers, and patients’ outcomes. *AF* atrial fibrillation, *ECP* emergency care practitioner, *ECV* electrical cardioversion, *SR* sinus rhythm

Between December 12, 2018 and January 15, 2020, 100 patients in sinus rhythm after a previous successful in-hospital, propofol-supported ECV for symptomatic persistent AF were screened for participation (Fig. 1). Potential study participants were eligible for inclusion if a repeat ECV for a recurrence was deemed indicated by the attending cardiologist. Other inclusion criteria were: age between 20 and 75 years, BMI < 35 kg/m^2^ and demonstrated adherence to oral anticoagulation. Patients were required to have an American Society of Anaesthesiologists (ASA) score below 3 [10]. Patients with significant structural heart disease, known sick sinus syndrome or other arrhythmias, or those with a pacemaker or implantable cardioverter-defibrillator were excluded. Additionalexclusion criteria included severe comorbidities such as kidney dysfunction (eGFR ≤ 30 ml/min), pulmonary dysfunction (FEV1 < 1 litre), and active malignancy.

Out of 27 eligible patients, 25 provided informed consent (Fig. 1). To ensure safe cardioversion, the ambulance service’s ECP examined the patients’ homes for accessibility, hygiene, adequate space and lighting, electrical interference and explosive sources (e.g., oxygen tanks), electricity network stability, and the presence of an adequate informal caregiver overnight, usually a relative. Consenting patients were instructed to call the ECP if they experienced a recurrence of AF. At the time of recurrence—and well before the actual cardioversion—potential intercurrent conditions such as heart failure or acute coronary syndrome were excluded. In addition, the indication for the repeat cardioversion was reconfirmed by the attending cardiologist.’

Of the 25 consenting patients, six developed one or two recurrences of AF and underwent ECV at their homes (Tab. 1). Patients were instructed to fast at least 6 h before the scheduled cardioversion time. To ensure optimal use of resources, the procedures were planned in downtime windows with peak availability of ambulance, ECV team members and remote supervision by a cardiologist. One hour before the intervention, the ECP arrived at the patient’s home to confirm AF using a portable Corpuls‑3 device (Corpuls® BV, Hellevoetsluis, NL), and to perform point-of-care tests to check potassium and INR level (if vitamin K antagonists were used). If all checks were in order, the cardiologist’s approval was acquired by telephone consultation.

**Table 1:** Baseline clinical characteristics of the 6 study patients who underwent one or more electrical cardioversions at home.

Nr	Age (years)	Sex	BMI	Underlying conditions	CHA2DS2-VASc score	AF history (years)	Previous clinical ECVs (*n*)	AF free pre-ECV@home (days)
1	53	M	29	HT, MVP	1	3	2	205
2	55	M	24	Lone AF, PVIs	0	12	8	187
3	59	M	26	AVR, CAD	1	18	13	483
4	61	M	28	OSAS	0	2	2	445
4r	62							134
5	72	F	34	HT, DM	4	6	4	609
6	74	M	25	HT, PVI	2	17	6	81
6r	74							201

*BMI* body mass index, *AF* atrial fibrillation, *AVR* aortic valve replacement, *CAD* coronary artery disease, *DM* diabetes mellitus, *ECV@home* electrical cardioversion at home, *HT* hypertension, *MVP* mitral valve prolapse, *OSAS* obstructive sleep apnea, *PVI* pulmonary vein isolation, *r* repeat cardioversion (second in the study)

Thereafter, the ambulance driver and sedation nurse would arrive at the patient’s home. Although the ambulance team could manage most complications on-site using current national ambulance sector protocols, the ambulance’s presence was required to ensure hospital transfer if necessary. Patients were positioned on the ambulance stretcher—mostly in the patient’s living room—and connected to the Corpuls‑3 monitor; propofol sedation was then started. The stretcher is the natural treatment environment of the ECP, facilitated interventions in case of complications and was also used for ergonomic reasons (e.g. adjustable height and (anti‑)Trendelenburg position). ECV was performed using synchronised biphasic 200 Joules shocks. After successful cardioversion, return of consciousness (Aldrete score ≥ 9) [11] and stable vital signs were confirmed. The ambulance driver and sedation nurse then leave the scene. After one additional hour of stable consciousness, normal pulse, blood pressure, respiration, oxygen saturation, and carbon dioxide concentration, the ECP handed over care to the informal caregiver and debrief the supervising cardiologist. Follow-ups were performed by the ECP at 6 and 24 h, assessing AF recurrence (by symptoms and, an if indicated, ECG), complications (via questionnaire), and patient satisfaction (by asking preference for either procedure). An outpatient cardiologist visit followedat 6 weeks to evaluate the rhythm status and any complications. The complete protocol is presented in the Supplement. All data, including ECV outcomes, were recorded in the patient’s electronic medical record. Descriptive statistics were used to summarise patients’ characteristics and outcomes.

## Results

Pre-cardioversion home examinations were performed in all 25 consenting patients and did not yield any obstacles to cardioversion at home. Tab. 1 describes the characteristics patients who experienced a recurrence of AF and underwent ECV at home. One female participated in the study. All patients had a year-long AF history, and the AF-free interval before the home cardioversion averaged 293 ± 179 days (median 325 days). Tab. 2 shows procedural details of all 8 cardioversions in the 6 study patients. Home cardioversion was mostly performed either the day of recurrence or the day thereafter. Delays in 2 patients (#4 and #6) were due to inadequate anticoagulation and a 3-day patient delay, respectively. Both of these patients experienced a recurrence after 134 and 201 days, respectively, and underwent a second home cardioversion within the study period. In both cases, cardioversion was successfully performed on the day of the recurrence. Prior to cardioversion, all patients were on adequate oral anticoagulation, and potassium levels ranged between 3.8 and 4.3 mmol/L. The mean heart rate during AF before cardioversion was 111 ± 19 bpm. All patients converted to normal sinus rhythm after a single biphasic shock of 200 J. Following conversion, heart rate dropped to 66 ± 13 bpm, with no clinically significant bradycardias were observed. The time between arrival of the ECP and application of the first shock amounted was 68 ± 15 min, while the total procedure time at the patients’ homes—up to full recovery—averaged 136 ± 16 min. There were no acute complications, and no complications during 6‑weeks follow-up. All patients were satisfied with the procedure and would opt for home cardioversion rather than a clinical cardioversion in case of an eventual AF recurrence.

**Table 2:** Procedural details of all cardioversions at home.

Nr	Timing of ECV@home	Heart rate in AF (bpm)	Negative dromotropic drugs	AAD	Propofol (mg)	Time to 1st shock (hr:min)	Procedure duration (hr:min)
1	Same day	75	None	None	130	1:04	2:19
2	Same day	133	None	None	120	1:27	2:47
3	Next day	113	Betablocker	Sotalol	50	1:04	2:05
4	> 3 weeks*	116	Betablocker	Sotalol	100	0:45	1:50
4r	Same day	100	&	&	100	1:05	2:21
5	Same day	131	Betablocker	Sotalol	100	0:59	2:11
6	> 3 days#	118	None	None	70	1:08	2:14
6r	Same day	102	&	&	70	1:30	2:24

*ECV@home* electrical cardioversion at home, *AAD* antiarrhythmic drugs, *AF* atrial fibrillation, *r* repeat cardioversion (second in the study)

* For re-installing oral anticoagulation after previous discontinuation for chronic sinus rhythm

# Due to patient delay

& Patients were on same medication at second cardioversion in the study

## Discussion

This proof-of-concept study suggests that, in highly selected patients, cardioversion at home led by an emergency care practitioner—supported by a sedation nurse, with remote supervision by a cardiologist and a safety-net ambulance present at the scene—is feasible, safe and effective. Also, patients invariably preferred home cardioversion over cardioversion in-hospital. Home cardioversion is expected to reduce hospital care costs. Moreover, a short time between AF recurrence and restoration of sinus rhythm may improve quality of life [12] and decrease AF progression [13]. Randomised controlled trials may eventually show that home cardioversion—compared to in-hospital treatment—can keep patients out of hospital and contribute to sustainability of health care.

Whether home cardioversion is truly safe still needs to be established in future investigations, which are currently ongoing in the Netherlands. A strong prerequisite for safety is a highly specialised and dedicated team that maintains skills over time. Of note, Health Care Inspection in the Netherlands and an ad hoc sedation committee (see acknowledgements) reviewed and approved the protocol. This also provided a legal context for the present medical intervention without a physician on site with the patient.

The typical patient suitable for home cardioversion has an expected low recurrence rate and is clearly symptomatic during AF [14]. In addition, patients must have undergone at least one previous uneventful cardioversion, and the risk of complications during future interventions should be considered low. Tab. 1 represents such a typical patient, with a long AF-free interval after a previous cardioversion. However, if safety is proven, the indication for home cardioversion may be broadened and extended to other persistent AF patients beyond those included herein, or even to patients with an acute episode of symptomatic paroxysmal AF.

At present, it is unknown whether home cardioversion is cost-effective compared to cardioversion at a day care unit with central surveillance and a team serving several patients simultaneously. However, hospital-based facilities are relatively expensive compared to a comprehensively operating mobile care team. Also, the efficiency of day care may be more difficult to maintain in larger hospital organisations, for example, due to current difficulties in hiring enough personnel and developing off-peak-hour activities. On the other hand, with home cardioversion, travel time, checking safety of the environment before cardioversion, monitoring for one hour after the procedure and an ambulance stand-by, all generate extra costs. In view of the above, further studies in larger patient groups are clearly needed.

In the current study, a call from a patient experiencing a recurrence was treated as a prioritised semi-emergency. This explains the brief interval between recurrence and cardioversion (Tab. 2). However, we cannot deny that, as in other European countries, the availability of ambulances may become more strained resulting in longer response times, including for critical calls. This also applies to the availability of nurses who provide sedation. Nonetheless, we believe that incorporating hospital day care interventions into ambulance services will improve flexibility in the transmural care system. Hospital-at-home solutions may therefore offer promising alternatives to traditional in-hospital patient care, and are currently being tested in a variety of settings [15]. The present study may set an example, including for European cardiology practices, and my provide elements for Europe-wide regulations.

In conclusion, home cardioversion appears feasible and is generally preferred by patients over in hospital cardioversion.

## Data Availability

The data from this article cannot be shared for privacy reasons of participants in the study. The data, or a subset thereof, will be shared on reasonable request to the corresponding author.
